# Conditional removal of the canonical TGF-β1 signaling delays condylar cartilage degeneration induced by a partial discectomy in mice

**DOI:** 10.1371/journal.pone.0177826

**Published:** 2017-05-18

**Authors:** Jie Fang, Li Xiao, Rebecca Chen, Zhihe Zhao

**Affiliations:** 1 State Key Laboratory of Oral Diseases, National Clinical Research Center for Oral Diseases, West China Hospital of Stomatology, Sichuan University, Chengdu, Sichuan, China; 2 Department of Developmental Biology, Harvard School of Dental Medicine, Boston, MA, United States of America; 3 Department of Stomatology, Sichuan Academy of Medical Sciences and Sichuan Provincial People’s Hospital, Chengdu, Sichuan, China; China Medical University, TAIWAN

## Abstract

Recent emerging data indicate that the increase in the expression and activity of the transforming growth factor beta 1 (Tgf-β1) signaling may have detrimental effect to mature articular cartilage of knee joints. However, there is no information about whether or not this is the case in condylar cartilages. The objective of this study is to investigate the protein expression and activity of Tgf-β1 signaling in degenerative condylar cartilages. We also investigate biological effects of the conditional deletion of transforming growth factor receptor type II (*Tgfbr2*) in condylar cartilage of adult mice after a partial discectomy. Two mouse models of osteoarthritis (OA) were used to examine protein expressions of Tgf-β1 and p-Smad2/3 in condylar cartilages at early degenerative stages. In addition, cartilage specific *Tgfbr2*-deficient adult mice were subjected to a partial discectomy. The morphological condition of condylar cartilages was evaluated in mice at 4 and 12 weeks after the surgery. We found that protein levels of Tgf-β1 and p-Smad2/3 were increased in the degenerative condylar cartilage of the mouse models. The conditional removal of *Tgfbr2* in mature condylar cartilage significantly delayed the progressive progression of the cartilage degeneration induced by a partial discectomy. We conclude that the increase in the expression and activity of Tgf-β1 signaling may have detrimental effect to mature condylar cartilages. Therefore, inhibition of Tgf-β1 signaling may be able to protect condylar cartilages from being degraded in mature temporomandibular joints.

## Introduction

Condylar cartilage is the specialized avascular connective tissue covering the surface of the condyle of the mandible in temporomandibular joint (TMJ). The cartilage consists of fibroblast-like cells, chondrocytes and their associated extracellular matrix. The cells synthesize extracellular matrix components, including type I and type II collagens and proteoglycans that are major macromolecules in the matrix. The highly hydrated proteoglycans provide the structural basis of characteristic resilience of the cartilage. Collagens form a fine network that promotes the retention of proteoglycans and contributes to the mechanical function of the cartilage as a load-bearing tissue. Condylar cartilage degeneration is a gradual process characterized by the depletion of proteoglycans and the degradation of collagens. If the degenerative process continues without the replacement of extracellular matrix components, the degeneration eventually leads to osteoarthritis (OA). It is known that OA is one of the conditions that result in temporomandibular joint disease (TMD) [1–3]. The incidence of TMD caused by OA is increased in people older than 60 years of age [4]. The underlying molecular mechanisms of condylar cartilage degeneration are largely unknown. It has been reported that a number of factors can initiate the degeneration process, such as excessive mechanical force on a joint, a defect in condylar cartilage, injuries of joint structural elements and aging. Obviously understanding the molecular basis of condylar cartilage degeneration will provide invaluable information for the development of disease-modifying OA drugs (DMOADs) to treat TMDs.

Transforming growth factor beta 1 (TGF-β1) has been considered an anabolic factor to articular chondrocytes [5, 6]. However, recent emerging data suggest that the increase in the activity of TGF-β1 signaling may accelerate articular cartilage degeneration in adult knee joints. First, a human genetic study reports that a nucleotide change, 859C>T or 782C>T in SMAD-3, increases the level and activity of the TGF-β1 signaling in two human families associated with early-onset OA [7]. This is in agreement with the observation from studies showing that the level of TGF-β1 is significantly higher in human osteoarthritic tissues than in healthy articular cartilage [8, 9]. Second, a study by Zhen et al. demonstrates that the inhibition of Tgf-β1 signaling in mesenchymal stem cells of subchondral bone delays the development of OA in knee joints of adult mice [10]. Third, another independent study indicates that the protein level of Tgf-β1 is significantly increased in the articular chondrocyte of knee joints in mouse models of OA[11]. The conditional genetic removal of the transforming growth factor receptor type II (*Tgfbr2*) significantly attenuated articular cartilage degeneration in adult mouse knee joints [11]. Given the fact that the biochemical composition of condylar cartilage is different from that of articular cartilage, it is not known whether or not the Tgf-β1 signaling plays a significant role in mature condylar cartilage degeneration.

In this present investigation, we examined protein expressions of Tgf-β1 and p-Smad2/3, down-stream Tgf-β1 signaling molecules, in TMJ after a partial discectomy, including non-surgical contralateral side, and in TMJ of a genetic form of mouse OA model, collagen type XI haploinsufficiency (*Col11a1*^*+/-*^) mice. We also conditionally removed *Tgfbr2* from mature condylar cartilages in mice and then the mice were subjected to a partial discectomy to induce condylar cartilage degeneration. We examined condylar cartilages for evidence of morphological changes in *Tgfbr2*-deficiency mice and their wild-type littermates after the partial discectomy.

## Materials and methods

The experimental procedures for animals were performed following approval from Harvard Medical School Institutional Animal Care Committee (Permit Number: 04446). This study was performed in accordance with the recommendations in the Guide for the Care and Use of Laboratory Animals of the National Institutes of Health. All efforts were made to minimize suffering.

### Partial discectomy of TMJ in mice

The detail of the surgical procedure has been described in the previous study [12]. Briefly C57BL/6j mice at the age of 3 months were used for the surgery. After mouse was anesthetized intraperitoneally with 90 mg ketamine/kg mouse body weight and 10 mg Xylazine/kg mouse body weight, an incision was made over the left TMJ. The lateral part of the disc was removed. For sham surgery, TMJ underwent a similar surgical procedure without the cut of the disc. Mice were fed with powder food for a week immediately after the surgery and then with regular food for the remainder of the experiment.

### Generation of *Col11a1*^+/-^ mice

The OA-like changes of TMJ in heterozygous chondrodysplasia (*cho*/+) mice was characterized [13]. The genetic mutation of *cho/cho* mice is identified, a single nucleotide deletion in the gene for α1 chain of type XI collagen (*Col11a1*) [14]. The protein product of *Col11a1* was not detectable in *cho/cho* mice. Thus the mutation functionally knocked out *Col11a1* in *cho/cho* mice (*Col11a1*^-/-^), which was prenatally lethal. For genotyping *Col11a1*^+/-^ mice, direct DNA sequence was performed. A mouse genomic DNA was isolated from a 3 mm segment of tail. A regular PCR was performed with the forward primer 5’-GGTGTTGTCCTGGGTAAACAG-3’ and the reverse primer 5’- GCAGTCACCATTGTCTAATCATC-3’. A size of 528 base-pair DNA fragment was generated by PCR. A primer, 5’- AGCTAAGCCACTGAGGCACAA-3’, next to the forward primer was used for direct DNA sequencing of the PCR product. *Col11a1*^+/-^ mice and their wild-type littermates identified by the DNA sequence were separated and maintained under a daily schedule of 12 hours with light and 12 hours without light.

### Immunohistochemistry staining

Four randomly TMJ in each experimental group was selected for experiments. For analysis of the protein expression of Tgf-β1 and p-Smad2/3, eight to ten paraffin sections, evenly distributed throughout each joint, were used for immunohistochemistry staining. Sections were deparaffinized and quenched for endogenous peroxidase activity using a 1% hydrogen peroxide solution. The sections were incubated with a rabbit polyclonal antibody (1:200) against Tgf-β1 protein (Cat. No. 5559–100, BioVsion, www.biovision.com), or a rabbit polyclonal antibody (1:2,000) against p-Smad2/3 (Cell Signaling Technology, Danvers, MASS). After overnight incubation at 4°C, the sections were washed with PBS three times followed by the treatment with a biotinylated secondary antibody (goat anti-rabbit IgG) (1:250) at room temperature for 30 min. The sections were then washed three times with PBS. Color detection was performed with peroxidase substrate after incubation with a mixture of avidin and biotinylated HRP. Sections were counterstained with 0.2% Fast Green solution. Staining without a primary antibody was performed as a negative control.

### Conditional removal of *Tgfbr2* in condylar cartilages of adult mice

The mouse strain, *AgcCreERT2*^*+/-*^, which expresses the fusion protein of CreERT2 (Cre-recombinase with the modified human estrogen receptor) under the control of the endogenous aggrecan promoter was provided by Dr. Stepnen Henry at MD Anderson Cancer center, TX. The floxed *Tgfbr2* mouse strain (*Tgfbr2*^f/f^) was obtained from National Cancer Institute, NIH (mouse strain number 01XN5). By crossing heterozygous *AgcCreERT2*^*+/-*^ mice with homozygous *Tgfbr2*^*f/f*^ mice, double heterozygous mice, *AgcCreERT2*^*+/-*^*;Tgfbr2*^*+/f*^, were obtained. Then, *AgcCreERT2*^*+/-*^*;Tgfbr2*^*f/f*^ mice were generated by crossing the double heterozygous mice. *AgcCreERT2*^*+/-*^*;Tgfbr2*^*f/f*^ mice at the age of 8 weeks old were injected intraperitoneally with tamoxifen at 2 mg/10 g body weight/daily for 8 consecutive days or injected with sunflower seed oil as control. For mice genotyping, regular PCRs were performed. A pair of PCR primers for *AgcCreERT2*^*+/-*^ was: forward 5’-TAACTACCTGTTTTGCCGGG-3’ and reverse 5’-GTCTGCCAGGTTGGTCAGTAA-3’. A pair of PCR primers for *Tgfbr2*^*f/f*^ was: forward 5’-TAAACAAGGTCCGGAGCCCA-3’ and reverse 5’-ACTTCTGCAAGAGGTCCCCT-3’.

### The removal efficiency of *Tgfbr2* in *AgcCreERT2*^*+/-*^*;Tgfbr2*^*Δ/Δ*^ mice.

The experimental procedure to generate the conditional deletion of *Tgfbr2* in mature condylar cartilage of TMJ in mice (*AgcCreERT2*^*+/-*^*;Tgfbr2*^*Δ/Δ*^) has been described in previous publication [12]. Briefly, *AgcCreERT2*^*+/-*^*;Tgfbr2*^*f/f*^ mice at the age of 8 weeks old were injected intraperitoneally with tamoxifen at 2 mg/10 g body weight/daily for 8 consecutive days or were injected with sunflower seed oil as control. Articular cartilages of knee joints were collected for isolation of the genomic DNAs. Since the exon 2 of *Tgfbr2* was floxed, real-time PCR was performed for the quantitative measurement of the exon 2 in aggrecan-expressing cells in the cartilages. *Tgfbr2* was removed in cells without the exon 2 of *Tgfbr2* (*AgcCreERT2*^*+/-*^*;Tgfbr2*^*Δ/Δ*^). The cartilage oligomeric matrix protein was used as an internal control. A pair of primers for the exon 2 of *Tgfbr2* was: forward 5’-AACAGTGATGTCATGGCCAG-3’ and reverse 5’-CAGACTTCATGCGGCTTCTC-3’. A size of 155-bp PCR product was generated by the primers. A pair of primers for the *cartilage oligomeric matrix protein* was: forward 5’-ACCCACAACAGGCACATT-3’ and reverse 5’-TCAGTCATAGGAAGCAGG-3’. A size of 142-bp PCR product was generated by the primers.

### Histology

The experimental procedure for histology has been published [13]. Briefly, at 4 and 12 weeks following the surgery, 5 heads of the discectomy mice and 5 heads of the sham mice were collected. All of the heads were fixed in 4% paraformaldehyde for 6 hours at room temperature. After the decalcification in Morse’s solution, each head was cut in half along the mid-sagittal plane and each half of the head was then embedded in paraffin. Serial sections were cut at a thickness of 6 μm. It took about 100 sections to cover a mouse TMJ from anterior to posterior. Every tenth section was collected for evaluation of morphological condition in each TMJ.

### Evaluation of articular cartilage conditions by a scoring system

The pathologic condition of articular cartilages was evaluated by a scoring system designed to assess the histology of OA in mouse joints; the system is recommended by the osteoarthritis research society international (OARSI) histopathology initiative [15]. The scores are: 0 for normal mouse articular cartilage, 0.5 for increase of Safranin-O without structural changes, 1 for small fibrillations without loss of cartilage, 2 for vertical clefts down to the layer immediately below the superficial layer and some loss of surface lamina, 3 for vertical clefts/erosion to the calcified cartilage extending to <25% of the articular surface, 4 for vertical clefts/erosion to the calcified cartilage extending to 25–50% of the articular surface, 5 for vertical clefts/erosion to the calcified cartilage extending to 50–75% of the articular surface, 6 for vertical clefts/erosion to the calcified cartilage extending >75% of the articular surface. We notice that the increase (not decrease) in Safranin-O staining is the earliest pathological sign in condylar cartilages. We, therefore, use the score 0.5 to represent this morphological change.

### Statistical analysis

Eight to ten sections, which were evenly distributed in each joint, were examined and scored. The score from the section of the worst condition was selected to represent that joint. Since there were five animals in an experimental group, thus 5 single scores were obtained for each group. Then, an average score was calculated from the five single scores for each group.

We used t-tests with a significance level of 0.05 to determine whether a significant difference between any two scores was present. To determine sample size in this study, we performed a pilot study on the effect of the *Tgfbr2*-deficiency. From the results, we concluded that a sample size of minimum 5 is required to achieve the specified confidence interval (95%) with at least 50% reduction of the score in the treatment group.

## Results

### Increases in protein expression of Tgf-β1 in degenerative condylar cartilages of adult mice

We examined protein level of Tgf-β1 in condylar cartilages in mice at 4 weeks following the partial discectomy and in *Col11a1*^+/-^ mice at the age of 3 months old. With regard to the morphological changes, increase in Safranine O staining for proteoglycans was seen throughout the entire condylar cartilage and in the articular cartilage of the fossa in both the partial discectomy and *Col11a1*^+/-^ mice [12, 13]. This is one of the earliest characteristic appearances of the condylar cartilage degeneration. The over production of proteoglycans was also seen in the condylar cartilage in contralateral non-surgical TMJ of mice at 8 weeks post-discectomy. The distribution of proteoglycans was only in the deep zone of the condylar cartilages in wild-type control.

**Fig 1:**
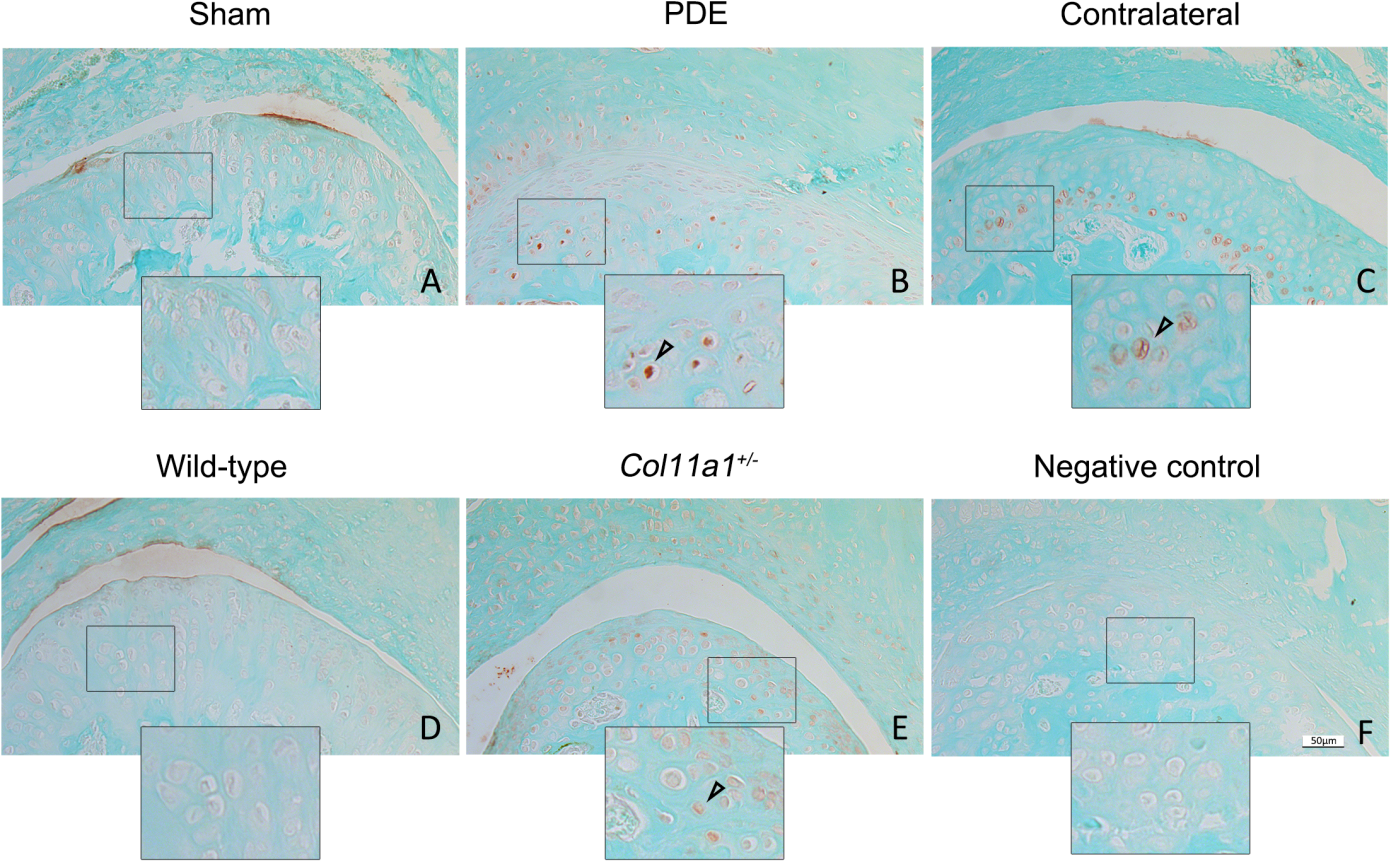
Increase in the protein expression of Tgf-β1 in condylar cartilages in mice. TMJ from discectomy and *Col11a1*^*+/-*^ mice were used for examination of the protein expression of Tgf-β1. The brown-color cells (see the cell in the inset) in discectomy (B) and *Col11a1*^*+/-*^ (E) mice indicate positive staining for Tgf-β1. The positive-staining cells were also seen in the condylar cartilage in contralateral non-surgical TMJ of mice at 8 weeks post-discectomy (C). The positive staining of the cells was predominantly distributed within the basal layer of the condylar cartilage. The positive staining cells were seen in all of discectomy and *Col11a1*^*+/-*^ mice. The location of the increased expression of Tgf-β1 was randomly throughout the basal layer of the condylar cartilages. In contrast, there were hardly the positive staining of cells for Tgf-β1 in sham surgery mice as control for discectomy mice (A) and wild-type mice as control for *Col11a1*^*+/-*^ mice (D). Staining without the primary antibody was used a negative control (F). (Bar = 50 μm).

We found that the protein expression of Tgf-β1 was increased in the partial discectomy and *Col11a1*^+/-^ mice (brown-color stained cells in Fig 1). The increased expression of Tgf-β1 was predominantly in the deep zone of the condylar cartilage. We did not detect the protein expression of Tgf-β1 in either the wild-type or the sham surgery control mice.

### Increases in protein expression of p-Smad2/3 in degenerative condylar cartilages of adult mice

**Fig 2:**
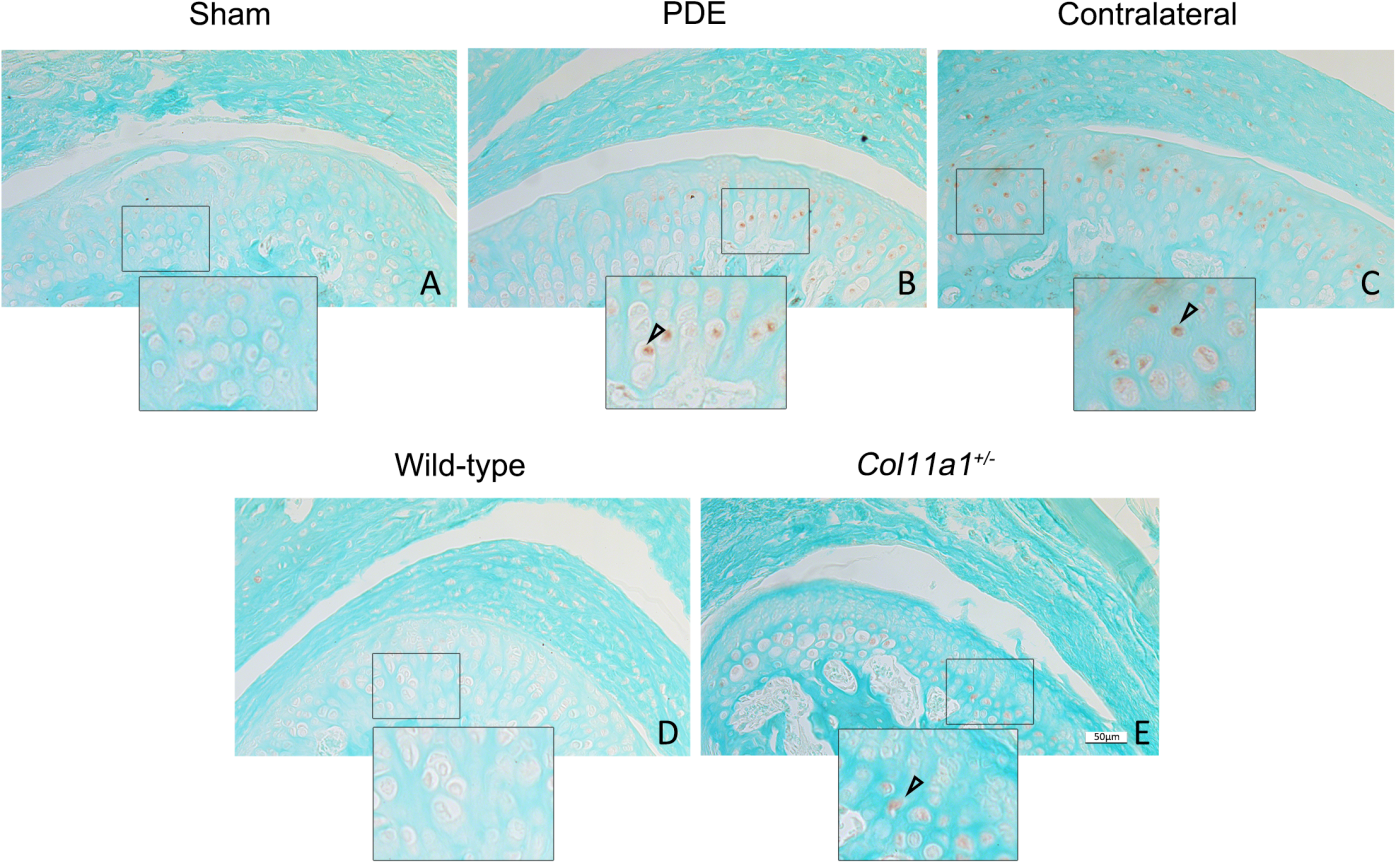
Increase in the protein expression of p-Smad2/3 in condylar cartilages in mice. There were p-Smad2/3 protein positive staining cells (brown-color staining) in the condylar cartilage of discectomy mice (B), *Col11a1*^+/-^ mice (E) and the contralateral non-surgical TMJ of mice (C). The expression patterns of p-Smad2/3 were similar to what were seen in the elevated expression of Tgf-β1 in the Fig 1 above. The positive-staining cells were seen within the basal layer of the condylar cartilages. There were no positive staining cells seen in sham control (A) and wild-type mice as control (D). (Bar = 50 μm).

Receptor-regulated SMADs 2 and 3 (R-SMAD2/3) are down-stream factors of the TGF-β1 canonical signaling pathway. We found that protein expression of p-Smad2/3 was increased in the partial discectomy and *Col11a1*^+/-^ mice (brown-color cells in Fig 2). The increased expression pattern of p-Smad2/3 was similar to that of Tgf-β1, which was predominantly in the deep zone of the condylar cartilage. We did not detect the protein expression of p-Smad2/3 in the control mice.

### The removal efficiency of *Tgfbr2* in aggrecan-expressing cells

We found that the exon 2 of the *Tgfbr2* was deleted in 86% of aggrecan-expressing cells in the articular cartilages [11]. The loss of the exon 2 resulted in a pre-mature stop codon immediately after the exon 1 of *Tgfbr2*. This indicates that *Tgfbr2* was removed in the majority of aggrecan-expressing cells in cartilages of adult joints in *AgcCreERT2*^*+/-*^*;Tgfbr2*^*f/f*^ mice with the treatment of tamoxifen.

### Deceleration of the progressive process of condylar cartilage degeneration in cartilage specific *Tgbr2*-deficient mice

**Fig 3:**
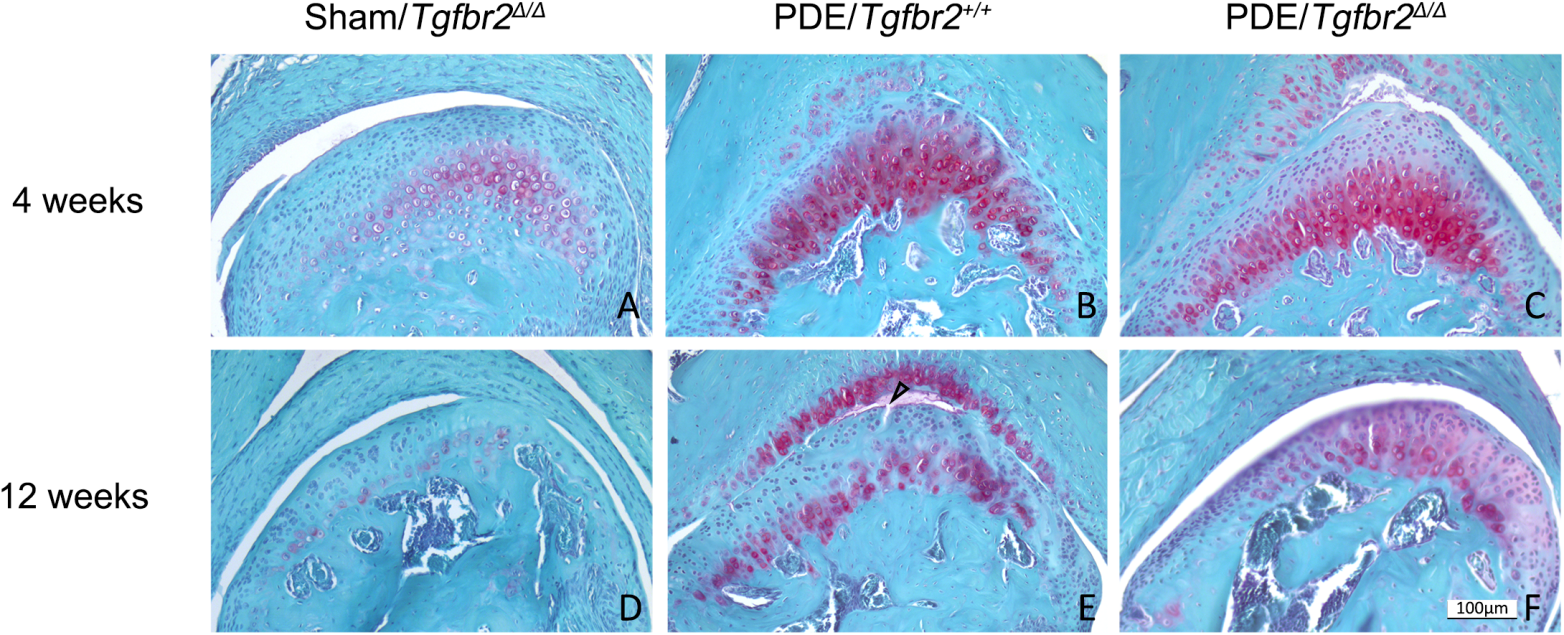
Morphology of condylar cartilages of mice after a partial discectomy. At 4 weeks after the discectomy, increase in Safranine O staining for proteoglycans was seen throughout the entire condylar cartilage and in the articular cartilage of the fossa in the partial discectomy of wild-type mice (*Tgfbr2*^*+/+*^) (B). However, the distribution of proteoglycans was present in the deep zone of the condylar cartilages of *Tgfbr2*-deficient mice (C). At 12 weeks after the surgery, fibrillation was seen (the arrow) in the condylar cartilage of *Tgfbr2*^+/+^ littermates (E). However, there was only the localized absence of the proteoglycans observed in *Tgfbr2*-deficient mice (F). There were no morphological abnormalities observed in sham controls (A and D). This suggests that the degenerative process was delayed in the condylar cartilages of *Tgfbr2*-deficient mice following the surgery. (Bar = 100 μm).

To determine the effect of the genetic deletion of *Tgfbr2* on the condylar cartilage degeneration, we performed a partial discectomy on *Tgfbr2*-deficient mice. We found that the morphology of condylar cartilages remained similar among the sham surgery groups of mice at the ages of 4 and 12 weeks old. However, following the surgery, the *Tgfbr2*-deficient mice and their wild-type littermates exhibited significant disparities in the progressive process of the condylar cartilage degeneration. The progression towards OA was dramatically delayed in the *Tgfbr2*-deficient mice (Fig 3).

The condition of the condylar cartilage degeneration was evaluated with a modified Mankin scoring system. Mice at 4 weeks following sham surgery were used as a normal control (score = 0). At 4 weeks following the surgery, scores for *Tgfbr2*-deficient mice and their wild-type littermates were 0.20 and 0.60, respectively. At 12 weeks following surgery, scores were 0.50 for *Tgfbr2*-deficient mice and 2.60 for the wild-type littermates. There were significant differences among the scores of these groups at each time point after the surgery, see Table 1.

**Table 1:** Evaluation of condylar cartilage condition in mouse TMJ.

Time points after PDE	Mean±SD		*p-*value
	*Tgfbr2*^*+/+*^	*Tgfbr2*^*Δ/Δ*^	
**4 weeks**	0.60 ± 0.18	0.20 ± 0.22*	0.0182
**12 weeks**	2.60 ± 0.45	0.50 ± 0.29**	0.0001

PDE, partial discectomy. The average scores (mean) in each group were based on 5 individual scores (n = 5). *Tgfbr2*^Δ/Δ^ indicates mice without *Tgfbr2*. There was a significant difference between the two groups of mice at 4 weeks

* p<0.05 (t-test). There was also a significant difference between the two groups of mice at 12 weeks

** p<0.01 (t-test).

## Discussion

### Increase in protein expressions of Tgf-β1 and p-Smad2/3 in degenerative condylar cartilages of adult mice

We examined expression profiles of Tgf-β1 and p-Smad2/3 in condylar cartilages of two mouse models of OA. The first model was early-onset OA of TMJ induced by a partial discectomy. This model revealed a typical pattern of condylar cartilage degeneration, starting at 4 weeks following the discectomy. Morphological analysis showed the over production of proteoglycans in TMJ of mice at 4 weeks post-discectomy, which was one of the earliest characteristic appearances of condylar cartilage degeneration. The increased production of proteoglycans was also found in the condylar cartilage in contralateral non-surgical TMJ of mice starting at 8 weeks post-discectomy [16]. This suggests the progressive progression of the cartilage degeneration on the contralateral side was present, but slower, compared to the discectomy side. The second model was condylar cartilage degeneration in *Col11a1*^+/-^ mice. The *Col11a1*^+/-^ mice developed normally with the exception of early-onset condylar cartilage degeneration. Histological studies showed that the proteoglycans were more diffused by spread over the entire layer of condylar cartilage in *Col11a1*^+/-^ mice at the age of 3 months.

Results from our immunohistochemistry staining demonstrated that the protein expression of Tgf-β1 was not detected in the condylar cartilage of wild-type mice. This indicated that the protein expression level of Tgf-β1 is, if any, very low in normal condylar cartilages. However, the expression and activity of Tgf-β1 signaling was significantly increased in the cartilage at early degenerative stage in both mouse models of OA. We, for the first time, found that the expression of Tgf-β1 was increased in the condylar cartilage of an injurious model of OA. The increase in the expression of Tgf-β1 in the condylar cartilage in *Col11a1*^+/-^ mice was consistent with the observation from another independent report [17]. In the line with our present investigation, the protein expressions of Tgf-β1 and p-Smad2/3 were also elevated in the articular cartilage of a surgery mouse model of OA[11].

The non-detection of Tgf-β1 by immunohistochemistry staining in mature condylar cartilage of wild type mice in this study indicated that there might not have significant biological effect of Tgf-β1 in the maintenance of condylar cartilage hemostasis in mice. We noticed that the increase in the expression and activity of Tgf-β1 signaling was associated the over production of proteoglycans in the condylar cartilage. This is also in line with a study reporting that the increased expression Tgf-β1 in adult mouse knee joints by adenovirus expressing vector results in the over production of proteoglycans, hyperplasia of synovium and chondro-osteophyte formation, which eventually leads a normal knee joint to become a OA joint in mice [18]. Thus the enhanced production of extracellular matrix molecules, such as proteoglycans, is not necessarily beneficial or physiological to mature cartilages. Results from several independent studies demonstrate that the increase in the amount of proteoglycan is one of the early pathological signs in the development of OA. The increased production of the proteoglycan does not prevent articular cartilages from being gradually degraded [16, 18]. The possible explanation for this is that mature articular cartilage is relatively quiescent and has no significant turnover, even with the occurrence of OA [19]. Thus, the over production of the proteoglycans alone may disrupt the homeostasis of mature cartilages.

### Condylar cartilage-protective effects by the conditional removal of *Tgfbr2* against the development of OA in adult mice

To determine whether or not the inhibition of Tgf-β1 signaling could decelerate the progressive progression of condylar cartilage degeneration, we conditionally deleted *Tgfbr2* in the mature condylar cartilage and then subjected the mice to the partial discectomy. We found that the progressive process of the condylar cartilage degeneration was significantly delayed in TMJ of the *Tgfbr2*-deficient mice. For example, we did not see the distribution of proteoglycans in the entire condylar cartilages in *Tgfbr2*-deficient mice at 4 weeks after the partial discectomy. We also did not observe the fibrillation in *Tgfbr2*-deficient mice at 12 weeks after the partial discectomy. In line of the observation from this study, another independent research group reports that the increase in the expression of Tgf-β1 in the subchondral bone of TMJ in mice causes early onset condylar cartilage degeneration [20]. Based on these results, we conclude that inhibiting activity of TGF-β1 signaling in mature condylar cartilages can, in fact, protect the cartilages from being degraded. This result also suggests that the increase in the expression and activity of Tgf-β1 signaling may have detrimental effects on mature condylar cartilages.

Many independent studies suggest that Tgf-β1 signaling is required for the development of joints. First, transgenic mice with a truncated Tgfbr2 develop early onset OA-like knee joints [21]. Second, Tgfbr2 is deleted in type II-collagen expressing cells in mice at the age of 2 weeks old. And the Tgfbr2-deficient mice exhibit the OA-like phenotype [22]. Third, Smad3 is deleted in every cell during mouse development. The Smad3-deficient mice also develop OA-like phenotypes [23]. Moreover, a human genetic study reports that a two-nucleotide deletion, 741-742del AT (a nonsense mutation), in SMAD-3 causes the early-onset OA in a human family [7].

How can we explain the attenuation of condylar cartilage degeneration by the removal of *Tgfbr2* in adult TMJ of mice? One possible explanation is that the TGF-β1 signaling is required in the development of joints. However, once a joint is formed, TGF-β1 signaling is no longer needed. For example, we did not observe any morphological abnormality in adult TMJ in cartilage specific *Tgfbr2*-deficient mice. However, the increase in the expression and activity of TGF-β1 signaling may lead to early onset condylar cartilage degeneration in adult TMJ. Another possible explanation is that Tgf-β1 signaling acts in a dosage dependent manner. Thus, the level of Tgf-β1 signaling above or below certain level will have biological effects on joints.

In summary, we believe that TGF-β1 is a pathogenic factor in the process of condylar cartilage degeneration in adult TMJ. Therefore, inhibiting activity of TGF-β1 signaling, not application of TGF-β1, should be considered in the treatment of TMD associated with condylar cartilage degeneration in adult patients.

## Supporting information

S1 Fig NC3Rs ARRIVE guidelines checklist. (PDF)
